# Differential Activation of Calpain-1 and Calpain-2 following Kainate-Induced Seizure Activity in Rats and Mice

**DOI:** 10.1523/ENEURO.0088-15.2016

**Published:** 2016-09-06

**Authors:** Jeff Seinfeld, Neema Baudry, Xiaobo Xu, Xiaoning Bi, Michel Baudry

**Affiliations:** 1 Graduate College of Biomedical Sciences, Western University of Health Sciences, Pomona, California 91766; 2 Department of Basic Medicine, Western University of Health Sciences, Pomona, California 91766

**Keywords:** calpain, hippocampus, interneurons, PTEN, seizure, spectrin

## Abstract

Systemic injection of kainate produces repetitive seizure activity in both rats and mice. It also results in short-term synaptic modifications as well as delayed neurodegeneration. The signaling cascades involved in both short-term and delayed responses are not clearly defined. The calcium-dependent protease calpain is activated in various brain structures following systemic kainate injection, although the precise involvement of the two major brain calpain isoforms, calpain-1 and calpain-2, remains to be defined. It has recently been reported that calpain-1 and calpain-2 play opposite roles in NMDA receptor-mediated neuroprotection or neurodegeneration, with calpain-1 being neuroprotective and calpain-2 being neurodegenerative. In the present study, we determined the activation pattern of calpain-1 and calpain-2 by analyzing changes in levels of different calpain substrates, including spectrin, drebrin, and PTEN (phosphatase and tensin homolog; a specific calpain-2 substrate) in both rats, and wild-type and calpain-1 knock-out mice. The results indicate that, while calpain-2 is rapidly activated in pyramidal cells throughout CA1 and CA3, rapid calpain-1 activation is restricted to parvalbumin-positive and to a lesser extent CCK-positive, but not somatostatin-positive, interneurons. In addition, calpain-1 knock-out mice exhibit increased long-term neurodegeneration in CA1, reinforcing the notion that calpain-1 activation is neuroprotective.

## Visual Abstract

**Figure:**
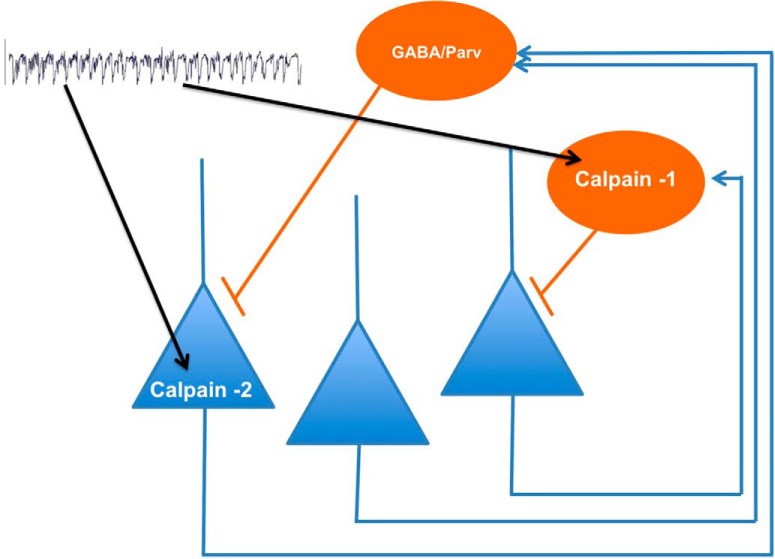

## Significance Statement

Seizure activity results in both short-term and long-term alterations in the structure and organization of neurons in hippocampus. While the activation of calpain has been well documented following kainic acid (KA)-induced seizures, there is no information regarding the roles of the two major calpain isoforms in the brain, calpain-1 and calpain-2, in the consequences of seizures. Here we report the surprising findings that, while calpain-2 is rapidly activated in all pyramidal cells of CA1 and CA3, calpain-1 activation is restricted to a small population of interneurons following systemic KA injection. Furthermore, KA-induced neurodegeneration in CA1 is exacerbated in calpain-1 knock-out mice, further supporting the notion that calpain-1 is neuroprotective and calpain-2 is neurodegenerative.

## Introduction

Systemic injection of kainic acid (KA) has been widely used to elicit seizure activities in both rats and mice, which can produce a rodent model of temporal lobe epilepsy (Nadler, 1981; Ben-Ari 1985; Lévesque et al., 2016). While the pathological sequelae of KA-induced seizures are well defined in rats, in mice they are highly dependent on the strain, with some strains being resistant to KA-induced neuronal degeneration and some being more susceptible (Schauwecker, 2002; Liu et al., 2012). Epileptic activity is associated with rapid morphological alterations in dendritic spines (Carpentier et al., 1991; González-Burgos et al., 2004) followed by profound reorganization of the hippocampal network and neuronal degeneration in CA3 and CA1, but not in the dentate gyrus (Sperk et al., 1983; Ben-Ari, 2001). Numerous cellular cascades are involved in KA-induced neurodegeneration, including calpain activation (Bi et al., 1996; Higuchi et al., 2005; Araújo et al., 2008; Feng et al., 2011), increased reactive oxygen species formation (Liang et al., 2000), microglial activation (Tooyama et al., 2002), and generation of neurotoxic lipid metabolites (Baran et al., 1987).

NMDA receptor activation following systemic KA-induced seizure activity is critical for seizure generation as well as for the subsequent neuronal damage (Berg et al., 1993; Brandt et al., 2003). Furthermore, the blockade of NR2B-containing NMDA receptors during seizure activity reduced neurodegeneration, and there is evidence that this effect is mediated by the extrasynaptic population of these receptors (Frasca et al., 2011). Recent evidence indicates that the activation of synaptic NMDA receptors results in calpain-1 activation and neuroprotection, while extrasynaptic NMDA receptor stimulation produces calpain-2 activation and neurodegeneration (Wang et al., 2013). While these two main calpain isoforms, calpain-1 and calpain-2, are ubiquitously distributed in the brain (Liu et al., 2008), their respective functions during epileptic activity and the resulting neurodegeneration have not been investigated. Calpain-2 can be activated via mitogen-activated protein kinase (MAPK) and extracellular signal-regulated kinase (ERK)-mediated phosphorylation, independent of calcium (Zadran et al., 2010), and ERK activation has also been observed after KA-induced seizures in adult rats (Otani et al., 2003). In addition, calpain-2 selectively degrades the phosphatase and tensin homolog (PTEN), resulting in mammalian target of rapamycin activation, stimulation of local protein synthesis (Briz et al., 2013), and cytoskeletal reorganization (Briz et al., 2015). Until recently, it was difficult to investigate the activation of each calpain isoform under different experimental conditions. Degradation of a preferred calpain substrate, spectrin, and analysis of the breakdown product generated by calpain cleavage has been widely used as a marker of calpain activation (Saido et al., 1993), but it does not differentiate calpain-1 and calpain-2. Likewise, there are no commercially available selective inhibitors for calpain-1 and calpain-2. The recent availability of calpain-1 knock-out (KO) mice has provided the opportunity to determine calpain activation following various patterns of electrical injury (Zhu et al., 2015) or after traumatic brain injury (Yamada et al., 2012). In addition, the recent discovery that PTEN is selectively degraded by calpain-2 and not by calpain-1 makes the degradation of PTEN a potential marker for calpain-2 activation (Briz et al., 2013). The present study was therefore directed at analyzing the activation patterns of calpain-1 and calpain-2 early after seizure initiation in order to understand their potential contributions to the short-term and long-term consequences of seizure activity. The results indicate that, while calpain-2 is rapidly activated in pyramidal neurons throughout CA1 and CA3, rapid calpain-1 activation is restricted to a small population of interneurons in CA1 and CA3. Moreover, the results strengthen the notion that calpain-1 activation is neuroprotective.

## Materials and Methods

Adult Sprague Dawley male rats or C57BL/6 [wild-type (WT) and calpain-1 KO mice on a C57BL/6 mouse background] male mice were kept under standard laboratory conditions with a 12 h light/dark cycle. To induce seizure activity, rats were injected with KA (12 mg/kg, i.p.; in saline) or saline (control). In some experiments, rats were also injected with either vehicle (1% DMSO in saline) or calpeptin (250 µg/kg) twice, at 1 d and 30 min before intraperitoneal injection with either saline or KA. For mice, seizure activity was induced by three injections of KA (5 mg/kg, i.p.) at intervals of 20 min. Mice were videotaped for 1 h, and the videos were analyzed to determine latency to first seizures, duration of first seizures, and cumulative duration of stage 3–5 seizures. This protocol was previously shown to produce status epilepticus by both behavioral and electroencephalographic studies (Tse et al., 2014). Animal experiments were performed in accordance with the principles and procedures of the National Institutes of Health *Guide for the Care and Use of Laboratory Animals*, and all protocols were approved by local Institutional Animal Care and Use Committees. Following KA injections, animals were observed for behavioral alterations, and only those exhibiting seizures were further processed. Seizure behavior was characterized by the presence of repeated head nodding, wet-dog shaking, repeated unilateral and bilateral limb-scratching movements, and, in some animals, tonic–clonic forelimb movements. Rats were killed at 2 or 4 h after seizure onset, while mice were killed at 1 h or 7 d after seizure onset.

### Immunohistochemistry

Animals were deeply anaesthetized with phenobarbital by injection (50 mg/kg, i.p.) and perfused intracardially with freshly prepared 4% paraformaldehyde in 0.1 m phosphate buffer, pH 7.4. After perfusion, brains were removed and immersed in 4% paraformaldehyde at 4ºC for postfixation, then in 15% and 30% sucrose at 4ºC for cryoprotection. Thirty micrometer coronal sections were prepared from mice brains with a vibratome. Immunohistochemistry was performed on free-floating brain sections blocked for 1 h at room temperature in 10% normal goat serum diluted in 1× TBS, pH 7.4, and then incubated with the following primary antibodies: spectrin breakdown product (SBDP; a generous gift from Dr. T. Saito, RIKEN, Tokyo, Japan), PTEN (catalog #26H9, Cell Signaling Technology), drebrin (catalog #10140, Millipore), parvalbumin (catalog #7449, Santa Cruz Biotechnology), somatostatin (catalog #7819, Santa Cruz Biotechnology), and CCK (catalog #21617, Santa Cruz Biotechnology) overnight at 4 ºC. After washing with PBS, sections were incubated in fluorescently labeled secondary antibodies in PBS with 5% goat serum for 2 h at room temperature. Sections were washed again with PBS and immediately mounted on slides with Prolong Gold Mount with DAPI. Images were acquired using a Nikon C1 Confocal Laser-Scanning Microscope. Acquisition parameters were kept identical among different experimental groups. All immunostaining studies were performed in three to five independent experiments. The mean fluorescence intensity of identical regions of interest in three to four sections per animal was determined with ImageJ. The mean fluorescence intensity in sections from each animal was calculated, providing the value for this particular animal. Values from all of the experimental animals in each group were averaged and normalized to the mean values found in the corresponding control group.

Cell death was analyzed 7 d after KA injection in WT and calpain-1 KO mice by using immunohistochemistry with an antibody against NeuN, a neuron-selective marker. A similar procedure as the one described above was used to quantify neuronal density in various hippocampal subfields.

### Western blots

For Western blot studies, rats were killed by decapitation following anesthesia with halothane, and hippocampi were rapidly dissected and homogenized in cold lysis buffer (50 mm Tris- HCl, pH 7.4, 150 mm NaCl, 5 mm EDTA, 1 mm EGTA, 1 mm PMSF, 10 mm NaF, and 1 mm Na3VO4), containing protease inhibitor cocktail (Thermo Scientific). Fifteen to twenty micrograms of proteins were loaded onto 8-15% SDS gels, separated by gel electrophoresis, and transferred onto polyvinylidene difluoride membranes. Membranes were blocked with 5% Odyssey blocking buffer for 1 h at room temperature, and incubated overnight in PTEN and drebrin primary antibodies at 4°C. After washing to remove free primary antibodies, membranes were incubated in fluorescent secondary antibodies for 30 min at 20°C. After a final wash, membranes were scanned by Odyssey image scanner (LI-COR Biosciences). Blots were stripped and reprobed with an antibody against actin, and the levels of PTEN and drebrin were normalized to those of actin. Results were averaged and normalized to the mean values in the control group.

### Statistics

All statistics were performed using GraphPad Prism version 4.03 software (GraphPad Software). One-way ANOVA was used for studies in rats, and two-way ANOVA was used for studies in mice to test whether the mean of each experimental group was significantly different from the others; and, if the overall *p* value was <0.05, then multiple comparisons between the experimental groups were performed using Tukey’s *post hoc* analysis with 95% confidence intervals.

## Results

### Changes in PTEN, drebrin, and spectrin in hippocampus 2 h after KA-induced seizure in 2-month-old rats

**Figure 1.**
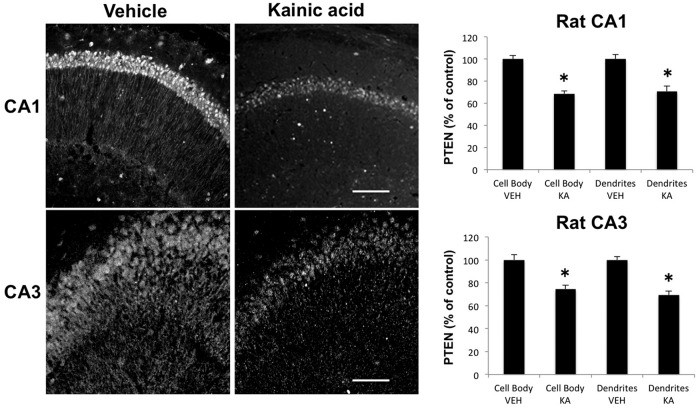
Effects of KA-induced seizure on PTEN levels in rat hippocampus. Rats were injected with KA (12 mg/kg, i.p.) and killed 2 h after seizure initiation. Coronal brain sections were processed for immunohistochemistry with an antibody against PTEN. Note the large decrease in labels in both cell body layer and apical dendrites of CA1 and CA3 pyramidal neurons. Fluorescence intensity was quantified, and the results were expressed as the percentage of values found in sections from saline-injected rats (mean ± SEM of four animals per group, with each animal value being the average of values measured in three to four sections). *p < 0.01, Student’s *t* test.

**Figure 2.**
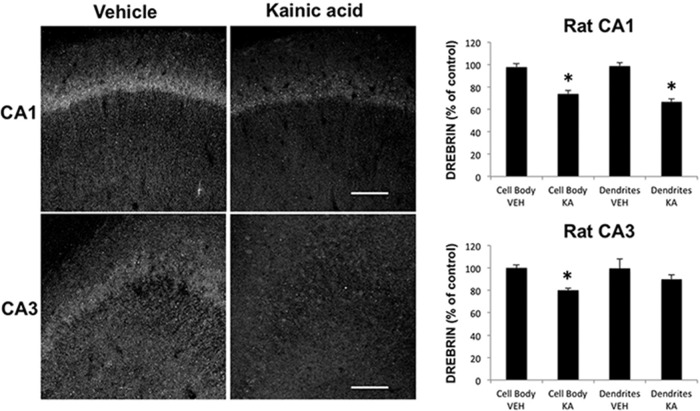
Effects of KA-induced seizure on drebrin levels in rat hippocampus. Rats were injected with KA (12 mg/kg, i.p.) and killed 2 h after seizure initiation. Coronal brain sections were processed for immunohistochemistry with an antibody against drebrin. Note the large decrease in label in both cell body layer and apical dendrites of CA1 and CA3 pyramidal neurons. Fluorescence intensity was quantified, and the results were expressed as the percentage of values found in sections from saline-injected rats (mean ± SEM of four animals per group with each animal value being the average of values measured in three to four sections). *p < 0.01, Student’s *t* test.

**Figure 3.**
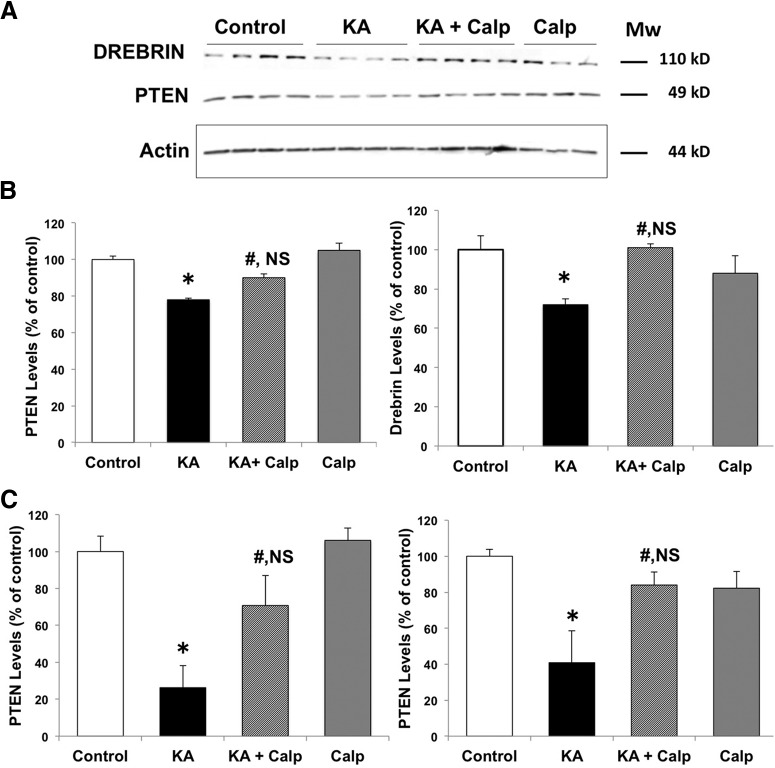
KA-mediated decrease in PTEN and drebrin is blocked by a calpain inhibitor. Rats were injected with KA (12 mg/kg, i.p.) and killed 2 h after seizure initiation. The calpain inhibitor calpeptin was injected 12 h and 30 min before KA injection. Hippocampi were dissected and homogenized, and aliquots of the homogenates were processed for immunoblot with PTEN and drebrin (actin was used as a loading control**). *A***, Western blots showing levels of PTEN, drebrin, and actin (PTEN and drebrin were labeled on the same blots, and the blots were reprobed with an anti-actin antibody, which was used to normalize the values for PTEN and drebrin). ***B***, Band intensity was quantified, and the results were normalized to actin levels. Results were then expressed as the percentage of controls, and are reported as the mean ± SEM of four independent experiments (three for the calpeptin-alone group). *p < 0.01, ANOVA followed by Tukey’s test. ***C***, PTEN immunohistochemistry was performed in other groups of rats similarly treated with KA and calpeptin. PTEN levels in CA1 and CA3 were quantified as described in Materials and Methods and normalized to the average values found in the respective control groups. Results are reported as the mean ± SEM of four to five animals per group. *p < 0.01, compared with controls; #*p* < 0.01, compared with the KA group; *p* = NS, not significantly different from control, ANOVA followed by Tukey’s test.

Previous studies indicated that KA-induced seizures in adult rats were associated with calpain activation, as assessed by changes in the specific SBDP generated by calpain-mediated cleavage, in selected neuronal populations at early time points after seizure onset, which spread throughout the pyramidal cells of CA3 and CA1 over the following 5 d (Bi et al., 1996). Because spectrin is cleaved by either calpain-1 or calpain-2, while PTEN is selectively cleaved by calpain-2, we first analyzed changes in PTEN 2 h after KA injection to assess calpain-2 activation. In contrast to the previously reported changes in SBDP, KA-induced seizures were associated with a widespread decrease in PTEN immunoreactivity (IR) throughout pyramidal neurons of CA1 and CA3 (Fig. 1; Table 1, lines 2–4). No changes in PTEN-IR were observed in granule cells of the dentate gyrus. It was recently reported that drebrin was also a calpain substrate (Chimura et al., 2015), and it was therefore of interest to study changes in drebrin levels in hippocampus following KA-induced seizures. Adjacent sections to those used for PTEN staining were stained with drebrin antibodies (Fig. 2; Table 1, lines 7–10). Drebrin was detected in both cell bodies and dendrites throughout the hippocampus in control rats. Drebrin-IR levels were also significantly decreased in CA1 and CA3 2 h after KA-induced seizures, and even more so at 4 h after seizure initiation (data not shown). Like PTEN, drebrin-IR was not modified in granule cells of the dentate gyrus. To further verify that the decrease in PTEN-IR and drebrin-IR was due to calpain activation, rats were pretreated with the calpain inhibitor calpeptin 1 d and 30 min before KA injection. Calpeptin is a cell-penetrating calpain inhibitor that binds to the active site of calpain, and reversibly inactivates both calpain-1 and calpain-2 (Tsujinaka et al., 1988). It has been shown that intraperitoneal injection of calpeptin inhibits inflammation, cell death, and axonal damage in animal models of multiple sclerosis, suggesting that calpeptin enters the CNS (Guyton et al., 2010). Rats were killed 2 h after seizure onset, and changes in PTEN and drebrin in hippocampus were analyzed by Western blots (Fig. 3A). Treatment with calpeptin significantly prevented KA-induced decreases in levels of both drebrin and PTEN **(** Fig. 3B; Table 1, lines 12–13**)**. Immunohistochemistry was also performed in additional groups of rats, and indicated that the loss of PTEN staining in both cell bodies and dendritic fields in CA1 and CA3 following KA injection was significantly reduced by calpeptin pretreatment (Fig. 3C; Table 1, line 14).

**Table 1:** Statistical table showing the data structure, the type of analysis, and the power

Line number	Parameter	Data structure	Type of test	Power
2	RATS CA1 PTEN cell bodies	Normal distribution	Unpaired *t* test	0.0001
3	RATS CA1 PTEN dendrites	Normal distribution	Unpaired *t* test	0.0013
4	RATS CA3 PTEN cell bodies	Normal distribution	Unpaired *t* test	0.0014
5	RATS CA3 PTEN dendrites	Normal distribution	Unpaired *t* test	0.0002
6				
7	RATS CA1 DREBRIN cell bodies	Normal distribution	Unpaired *t* test	0.001
8	RATS CA1 DREBRIN dendrites	Normal distribution	Unpaired *t* test	0.0001
9	RATS CA3 DREBRIN cell bodies	Normal distribution	Unpaired *t* test	0.0046
10	RATS CA3 DREBRIN dendrites	Normal distribution	Unpaired *t* test	0.3576
11				
12	RATS WB PTEN	Normal distribution	One-way ANOVA	0.001
13	RATS WB DREBRIN	Normal distribution	One-way ANOVA	0.0272
14	Rats CA1 and CA3 PTEN	Normal distribution	One-way ANOVA	0.01
15	RATS CA1 SBDP cell bodies	Normal distribution	Unpaired *t* test	0.0293
16	RATS CA1 SBDP dendrites	Normal distribution	Unpaired *t* test	0.0579
17	RATS CA3 SBDP cell bodies	Normal distribution	Unpaired *t* test	0.0405
18	RATS CA3 SBDP dendrites	Normal distribution	Unpaired *t* test	0.003
19				
20	MICE CA1 DREBRIN	Normal distribution	Two-way ANOVA, not RM	
21			Interaction	0.0047
22			Row factor	0.2943
23			Column factor	0.0001
24	MICE CA3 DREBRIN	Normal distribution	Two-way ANOVA, not RM	
25			Interaction	0.1237
26			Row factor	0.0086
27			Column factor	0.0001
28	MICE CA1 PTEN	Normal distribution	Two-way ANOVA, not RM	
29			Interaction	0.05
30			Row factor	0.2028
31			Column factor	0.0001
32	MICE CA3 PTEN	Normal distribution	Two-way ANOVA, not RM	
33			Interaction	0.0295
34			Row factor	0.373
35			Column factor	0.0001
36	MICE CA1 SBDP	Normal distribution	Two-way ANOVA, not RM	
37			Interaction	0.0001
38			Row factor	0.0001
39			Column factor	0.0001
40	MICE CA3 SBDP	Normal distribution	Two-way ANOVA, not RM	
41			Interaction	0.0005
42			Row factor	0.0052
43			Column factor	0.0001
44	MICE CA1 cell density	Normal distribution	Two-way ANOVA, not RM	
45			Interaction	0.2278
46			Row factor	0.0026
47			Column factor	0.0006
48	MICE CA3 cell density	Normal distribution	Two-way ANOVA, not RM	
49			Interaction	0.496
50			Row factor	0.126
51			Column factor	0.0001
52	MICE hilus cell density	Normal distribution	Two-way ANOVA, not RM	
53			Interaction	0.6033
54			Row factor	0.138
55			Column factor	0.0008

**Figure 4.**
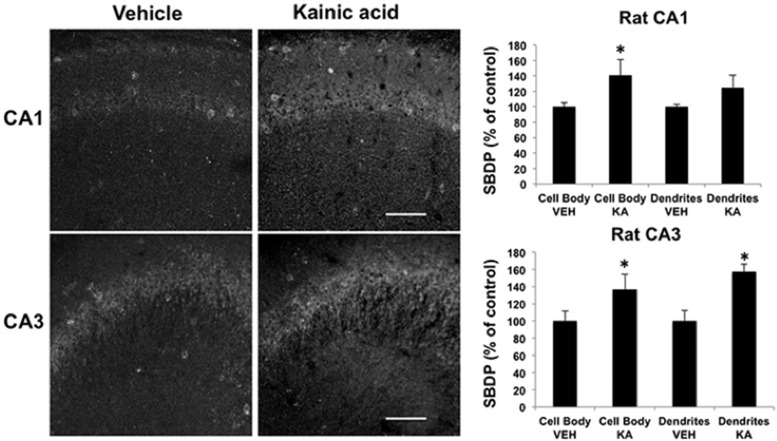
Effects of KA-induced seizure on spectrin truncation in rat hippocampus. Rats were injected with KA (12 mg/kg, i.p.) and killed 2 h after seizure initiation. Coronal brain sections were processed for immunohistochemistry with an antibody against calpain-mediated SBDP. Note the very restricted increase in SBDP in what looks like interneurons in CA1. In CA3, increased SBDP staining appears to take place in pyramidal neurons. Fluorescence intensity was quantified, and the results were expressed as the percentage of values found in sections from saline-injected rats (mean ± SEM of four animals per group with each animal value being the average of values measured in three to four sections). *p < 0.01, Student’s *t* test.

As mentioned above, immunohistochemistry with an antibody against SBDP was used to analyze calpain activation following KA injection in adult rats (Bi et al., 1996). We repeated this analysis but used a different antibody against SBDP. As was previously reported, increased SBDP staining was found 2 h after KA injection in sparse populations of interneurons in CA1 (Fig. 4A; Table 1, lines 15–16). In CA3, low levels of staining were observed in stratum pyramidale and in stratum radiatum. Quantitative analysis of the changes in immunoreactivity indicated that increases in SBDP levels were significant in both stratum pyramidale and in stratum radiatum (Fig. 4B; Table 1, lines 17–18). When comparing the decrease in PTEN staining and the increase in SBDP staining, it was clear that these two types of changes occurred in different cell populations (compare Figs. 1, 4). Considering the selectivity of calpain-2 for PTEN, these results suggested that calpain-2, but not calpain-1, was widely and rapidly activated following seizure initiation, while calpain-1 was activated only in restricted cell populations; moreover, the results also suggested that spectrin was cleaved by calpain-1 but not by calpain-2 under these conditions.

### Changes in PTEN, drebrin, and spectrin in hippocampus 1 h after KA-induced seizure in 2-month-old WT and calpain-1 KO mice

**Figure 5.**
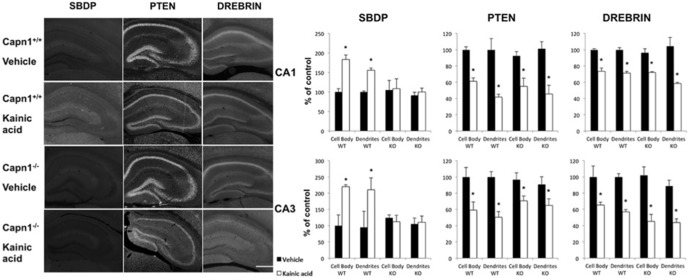
Effects of KA-induced seizures on hippocampal PTEN, drebrin, and SBDP in WT (Capn1^+/+^) and calpain-1 KO mice. WT and calpain-1 KO (Capn1^−/−^) mice were injected with KA as described in Material and Methods and were killed 1 h after seizure onset. Coronal brain sections were processed for immunohistochemistry with antibodies against SBDP, PTEN, or drebrin (left). Right, Images similar to those shown in left panels were processed for quantitative analysis of mean fluorescence intensity in different subfields of hippocampus. Fluorescence intensity was quantified, and the results were expressed as the percentage of values found in sections from saline-injected rats (mean ± SEM of four animals per group with each animal value being the average of values measured in three to four sections). *p < 0.01, Student’s *t* test.

**Figure 6.**
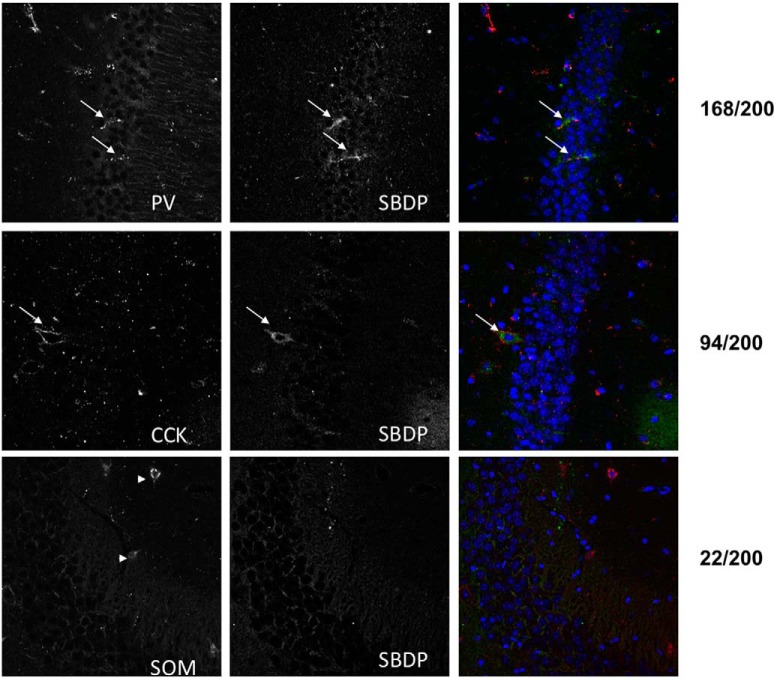
KA-induced seizures activate calpain-1 in parvalbumin- and CCK-containing, but not somatostatin-containing, interneurons in hippocampus. WT mice were injected with KA as described in Materials and Methods and were killed 1 h after seizure onset. Coronal brain sections were processed for immunohistochemistry with antibodies against SBDP, CCK, somatosatin (SOM), or parvalbumin (PV). Left panels, Immunohistochemistry with the antibodies against the indicated neuropetides. Middle panels, Immunohistochemistry with an antibody against SBDP. Right panels, Merged images. Numbers on the right indicate the relative numbers of double-labeled interneurons for the three neuropeptides. Arrows point to neurons labeled with both SBDP and parvalbumin, and CCK and SBDP antibodies. Arrowheads point to interneurons labeled with somatostatin but not SBDP antibodies.

To further determine the roles of calpain-1 and calpain-2 in the events triggered by seizure activity, we repeated the same study in WT and calpain-1 KO mice. Systemic KA injection elicited a similar pattern of seizure activities in WT and calpain-1 KO mice **(** Table 2**)**, with mice reaching stage 4 seizures within 1-2 h after injection. KA injection produced a decrease in PTEN and drebrin similar to those in rats in both WT and calpain-1 KO mice (Fig. 5; Table 1, lines 20–35). Decreases in PTEN and drebrin were highly significant in both the cell bodies and dendritic fields in CA1 and CA3, with no obvious decrease in the dentate gyrus. For SBDP, the changes in WT mice were very similar to those observed in rats (i.e., SBDP levels were slightly increased in stratum pyramidale and stratum radiatum in CA1 and CA3; Fig. 5; Table 1, lines 36–43). High-magnification examination of SBDP staining strongly suggested that the increase in SBDP was present in interneurons. To further determine which populations of interneurons exhibited seizure-induced spectrin truncation, and thus calpain activation, we performed double staining with markers for interneurons expressing CCK, somatostatin, or parvalbumin (Fig. 6). Analysis of a large number of double-stained interneurons indicated that spectrin was being cleaved by calpain, mostly in parvalbumin-expressing interneurons, to a lesser extent in CCK-expressing interneurons, and in only a small fraction of somatostatin-expressing interneurons (with 84% of parvalbumin-positive interneurons also positive for SBDP, as opposed to 47% of CCK-positive and 11% of somatostatin-positive interneurons also positive for SBDP). In contrast, no increase in SBDP levels was observed in either stratum pyramidale or stratum radiatum of CA1 and CA3 in calpain-1 KO mice (Fig. 5). These results confirmed that spectrin was cleaved by calpain-1, but not by calpain-2, under these conditions.

**Table 2:** Characteristics of KA-induced seizures in WT and calpain-1 KO mice

	WT	Calpain-1 KO
Latency to first seizures (s)	2107 ± 298	2063 ± 207
Duration of first seizures (s)	568 ± 83	611 ± 91
Cumulative duration of stage 3–5 seizures (s)	1603 ± 24	1646 ± 34

WT and calpain-1 KO mice were injected with KA, as described in Materials and Methods, and the animals were videotaped for 1 h after the last injection. Videos were analyzed for quantifying (1) latency to first seizures, (2) duration of the first seizure bout, and (3) cumulative duration of stage 3–5 seizures. Results are reported as the means ± SEM of six to seven animals.

### Enhanced neuronal death following KA injection in calpain-1 KO mice

**Figure 7.**
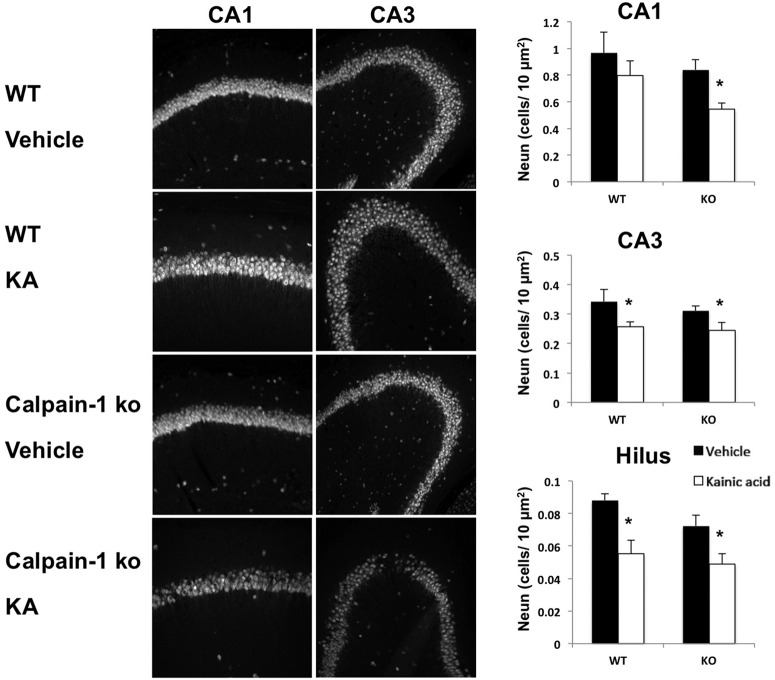
Effects of KA-induced seizures on neuronal density in hippocampus in WT and calpain-1 KO mice. WT and calpain-1 KO mice were injected with KA as described in Materials and Methods and were killed 7 d after seizure onset. Coronal brain sections were processed for immunohistochemistry with antibodies against NeuN. Cell densities in CA1, CA3, and the hilus of the dentate gyrus were quantified. Results are expressed as the number of cells per 10 µm^2^ and are reported as the mean ± SEM of four animals per group, with each animal value being the average of values measured in three to four sections. *p < 0.01, Student’s *t* test.

Calpain-1 and calpain-2 have been reported to play opposite functions in neuroprotection/neurodegeneration following the activation of synaptic versus extrasynaptic NMDA receptors, with calpain-1 activation being associated with neuroprotection and calpain-2 with neurodegeneration (Wang et al., 2013). It was therefore of interest to determine whether these different functions of calpain-1 and calpain-2 were also reflected in long-term neurodegeneration in hippocampus following KA injection. WT and calpain-1 KO mice were treated with KA and killed 7 d after injection. Pyramidal neurons in CA1 and CA3 were labeled with NeuN, and their numbers were quantitatively analyzed (Fig. 7). Previous results had shown that the C57BL/6 mice we used as the background strain for calpain-1 KO mice were resistant to KA-induced neuronal death (Ferraro et al., 1995). In agreement with these results, KA injection resulted in a small but not significant decrease in the density of neurons in CA1 and CA3 in WT mice. The density of neurons in CA1 was also slightly, but not significantly, lower in calpain-1 KO mice, compared with WT mice; in contrast, neuronal density in CA3 and in the hilus of the dentate gyrus was significantly lower in calpain-1 KO mice than in WT mice (Fig. 7; Table 1, lines 44–55). It was recently reported that calpain-1 KO mice exhibit increased apoptosis during the postnatal period in many brain structures, and that adult calpain-1 KO mice have a 10% reduction in the number of cerebellar granule cells (Wang et al., 2016). KA injection resulted in a significant decrease in the density of neurons in CA1 in calpain-1 KO mice compared with WT mice. However, it did not further modify neuronal density in CA3 and the hilus.

## Discussion

The goal of the present study was to determine the pattern of activation of calpain-1 and calpain-2 in hippocampus following KA-induced seizure activity in order to study their potential roles in the short-term and long-term consequences of seizures. Two complementary approaches were used to differentiate calpain-1 and calpain-2 activation. We first used the recently discovered selective calpain-2-mediated truncation of PTEN to analyze the pattern of calpain-2 activation in hippocampus following KA-induced seizures. It had previously been shown that another calpain substrate, spectrin, was cleaved by calpain following KA injection in adult rats (Bi et al., 1996), and we confirmed these results in the current study. As previously reported, our results indicated that 2 h after KA injection, spectrin was cleaved by calpain in very restricted cell populations, including some interneurons and pyramidal neurons in fields CA1 and CA3. In contrast, levels of PTEN decreased throughout the cell body layers and the dendritic fields of CA1 and CA3. That this effect was due to calpain-mediated truncation was indicated by its blockade, which was achieved by pretreating the animals with a nonselective calpain inhibitor, calpeptin. These results would therefore suggest that spectrin was truncated by calpain-1 activation in a relatively small subset of neurons, while PTEN would be degraded by calpain-2 activation in a much broader population of neurons. Our results also confirmed that drebrin, a neuron-specific F-actin binding protein, is also a calpain substrate, and is degraded following KA-induced seizure activity, a finding corroborating results indicating that drebrin levels were decreased in a pilocarpine model of epilepsy (Ferhat, 2012). As the pattern of changes for drebrin was very similar to that for PTEN, we therefore suggest that drebrin was also cleaved by calpain-2 in the hours following seizure initiation. Interestingly, the levels of drebrin in dendritic spines have been correlated with spine size and PSD length (Kobayashi et al., 2007), and our results would suggest that the decrease in drebrin levels following KA injection could result in smaller dendritic spines. Drebrin has also been found to regulate microtubule entry into dendritic spines (Merriam et al., 2013), and decreases in drebrin could also be associated with decreased levels of microtubules in spines.

While calpain-1 activation requires calcium, calpain-2 can be activated via MAPK/ERK-mediated phosphorylation, independently of calcium (Zadran et al., 2010). The MAPK/ERK pathway is activated as early as 15 min after and until 6 h after KA-induced seizure in adult rats (Otani et al., 2003), while calcium accumulation begins at 4 h after and was most pronounced on the 7th day after KA-induced seizure (Sztriha et al., 1986). Thus, our results suggest that calpain-2 activation was the result of widespread ERK-mediated phosphorylation in CA1 and CA3 pyramidal neurons. The differential patterns of spectrin, PTEN, and drebrin degradation could reflect the differential temporal activation of calpain-1 and calpain-2. Alternatively, it is possible that spectrin, PTEN, and drebrin have differential subcellular localizations, and their differential degradation could result from the differential activation of calpain-1 and calpain-2 in these subcellular compartments. The results also raise the issue of what initially triggers calpain-1 and calpain-2 activation following KA injection, although, as previously mentioned, it is possible that calpain-2 activation is due to ERK-mediated phosphorylation. In particular, since seizure activity can be prevented by NMDA receptor antagonists, it is clear that NMDA receptors are activated by seizure activity. Since it was previously shown that the activation of synaptic NMDA receptors triggers calpain-1 activation, widespread calpain-1 activation would have been expected (see below).

As previously reported (Bi et al., 1996), no obvious spectrin truncation was observed in the dentate gyrus granule cells. Similarly, no changes in PTEN and drebrin were found in these cells. These results indicate that neither calpain-1 nor calpain-2 was activated by KA-induced seizure activity in these cells. The reason for this lack of activation is not clear at this point, but is consistent with the fact that no long-term loss of granule cells takes place following seizure activity. This lack of calpain activation in the dentate gyrus granule cells contrasts with the clear activation of immediate early genes in these neurons following many types of stimulation, including seizure activity, which has been widely reported (Steward et al., 2001). We also found clear changes in levels of the activity-regulated cytoskeletal protein Arc, in both granule cells and pyramidal cells 4 h after KA injection, but the increase was not prevented by calpain inhibition (data not shown).

In the second approach, we used calpain-1 KO mice as a tool to assess the specific contribution of calpain-2 following seizure activity. While these mice do exhibit a mild form of cerebellar ataxia and impairment in synaptic plasticity and learning and memory (Zhu et al, 2015; Wang et al., 2016), the duration and severity of seizures were similar in WT and calpain-1 KO mice, indicating that the various differences we observed were not due to changes in neuronal excitability between the two genotypes. We found that, as in the rat, KA-induced seizure activity resulted in a rapid decrease in PTEN and drebrin levels in fields CA1 and CA3 of both WT and calpain-1 KO mice, with no significant change in the dentate gyrus. Thus, these results completely support our interpretation that decreases in PTEN and drebrin levels in CA1 and CA3 are due to calpain-2-mediated degradation. While the pattern of changes in spectrin truncation appeared to be slightly different in rats and WT mice, the results obtained with the calpain-1 KO mice also completely validated our interpretation of the rat data, indicating that calpain-1 activation is responsible for spectrin degradation following KA injection. It was previously reported that calpain-1 activation following synaptic NMDA receptor stimulation is critical to trigger neuroprotective signaling cascades (Wang et al., 2013). In agreement with this finding, we found that KA treatment produced a more severe loss of pyramidal neurons, at least in field CA1, in calpain-1 KO mice than in WT mice.

It is thus tempting to propose that following KA-induced seizure activity, calpain-1 and calpain-2 are differently activated, and that calpain-1 activation, possibly resulting from synaptic NMDA receptor stimulation, triggers an early neuroprotective signaling cascade potentially in a small subset of neurons in hippocampus. At the same time, calpain-2 activation would trigger neurodegenerative cascades, leading ultimately to widespread neuronal damage in field CA1 and CA3. As previously reported, C57BL/6 mice are resistant to the long-term effects of systemic KA injection, and we did not observe a significant decrease in the density of pyramidal neurons in WT mice, which contrasts with the very large decrease in the number of pyramidal neurons in rats treated with systemic KA administration. Nevertheless, KA elicited a significant decrease in neuronal density in calpain-1 KO mice, suggesting perhaps that the restricted activation of calpain-1 in WT mice in parvalbumin-containing interneurons, and to a lesser extent in CCK-containing interneurons, could nevertheless be involved in the neuroprotection of CA1 and CA3 neurons. It has recently been reported that parvalbumin-containing interneurons are critical for pyramidal cell synchronization of firing and the emergence of hippocampal theta rhythm (Amilhon et al., 2015). It is tempting to speculate that, by protecting these interneurons from KA-mediated neurodegeneration, calpain-1 activation maintains a sufficient degree of inhibition throughout the hippocampal circuitry to limit the extent of neurodegeneration in the pyramidal cells they innervate. Lack of calpain-1 would then lead to their early degeneration, eliminating a major set of interneurons, thereby exacerbating neuronal death in pyramidal cells. It will be interesting to see the results of further studies comparing different strains of mice to address this point. The lack of calpain-1 activation in pyramidal neurons following seizure activity is still somewhat surprising. Interestingly, high-frequency stimulation in mouse hippocampal slices also did not trigger calpain activation, and the underlying mechanism was shown to be the activation of PKA and the resulting phosphorylation of both calpain-1 and calpain-2 at inhibitory sites (Zhu et al., 2015). Whether a similar mechanism takes place following seizure activity remains to be determined.

In conclusion, our results indicate that calpain-1 and calpain-2 are differentially activated in hippocampus following seizure activity, with calpain-1 activation being limited to a small population of interneurons, and possibly triggering neuroprotective events, while calpain-2 is widely activated in pyramidal neurons of CA1 and CA3, and triggers neurodegenerative cascades.

## Synthesis

The decision was a result of the Reviewing Editor Uwe Heinemann and the peer reviewers coming together and discussing their recommendations until a consensus was reached. A fact-based synthesis statement explaining their decision and outlining what is needed to prepare a revision is listed below. The following reviewers agreed to reveal their identity: Wytse Wadman

This is a revised manuscript. the authorsd followed many of the suggestions of the reviewers except for providing video EEG data.The issue has been raised that 3-4 animals per group may be too little. The limitations this raises are partially compensated by the fact that multiple slices/sections have been included. The data also support each other in the different sections of the Ms. I suggest that the authors show in one r two aniamls EEG data for the kainate experiment to test whether there were really seizures induced. Way out could be referencing to work by others using excactly the same strain age sex and protocol for kainate treatment.
